# Whole-Genome Analysis of Diversity and SNP-Major Gene Association in Peach Germplasm

**DOI:** 10.1371/journal.pone.0136803

**Published:** 2015-09-09

**Authors:** Diego Micheletti, Maria Teresa Dettori, Sabrina Micali, Valeria Aramini, Igor Pacheco, Cassia Da Silva Linge, Stefano Foschi, Elisa Banchi, Teresa Barreneche, Bénédicte Quilot-Turion, Patrick Lambert, Thierry Pascal, Ignasi Iglesias, Joaquim Carbó, Li-rong Wang, Rui-juan Ma, Xiong-wei Li, Zhong-shan Gao, Nelson Nazzicari, Michela Troggio, Daniele Bassi, Laura Rossini, Ignazio Verde, François Laurens, Pere Arús, Maria José Aranzana

**Affiliations:** 1 IRTA, Centre de Recerca en Agrigenòmica CSIC-IRTA-UAB-UB, Campus UAB, Bellaterra (Cerdanyola del Vallès), Barcelona, Spain; 2 Consiglio per la ricerca in agricoltura e l’analisi dell’economia agraria (CRA), Centro di Ricerca per la Frutticoltura, Roma, Italy; 3 Università degli Studi di Milano, DiSAA, Milan, Italy; 4 Centro Ricerche Produzioni Vegetali, Cesena (FC), Italy; 5 Research and Innovation Centre, Fondazione Edmund Mach (FEM), San Michele all'Adige (TN), Italy; 6 INRA, UMR 1332 de Biologie du Fruit et Pathologie, Villenave d’Ornon, France; 7 Univ. Bordeaux, UMR 1332 de Biologie du Fruit et Pathologie, Villenave d’Ornon, France; 8 INRA UR1052 GAFL, Domaine Saint Maurice, Montfavet, France; 9 IRTA, Estació Experimental de Lleida, Parc de Gardeny, Edifici Fruitcentre, Lleida, Spain; 10 IRTA, Estacio Experimental Mas Badia, La Tallada d'Empordà, Girona, Spain; 11 Zhenzhou Fruit Research Institute, CAAS, Zhengzhou, China; 12 Horticultural Institute, Jiangsu Academy of Agricultural Sciences, Nanjing, China; 13 Department of Horticulture, Zhejiang University, Hangzhou, China; 14 Parco Tecnologico Padano, Lodi, Italy; 15 INRA, UMR 1345 Institut de Recherche en Horticulture et Semences, Beaucouzé, France; 16 Université d’Angers, UMR 1345 Institut de Recherche en Horticulture et Semences, SFR 4207 QUASAV, PRES L’UNAM, Angers, France; 17 AgroCampus-Ouest, UMR 1345 Institut de Recherche en Horticulture et Semences, Angers, France; Wuhan Botanical Garden of Chinese Academy of Sciences, CHINA

## Abstract

Peach was domesticated in China more than four millennia ago and from there it spread world-wide. Since the middle of the last century, peach breeding programs have been very dynamic generating hundreds of new commercial varieties, however, in most cases such varieties derive from a limited collection of parental lines (founders). This is one reason for the observed low levels of variability of the commercial gene pool, implying that knowledge of the extent and distribution of genetic variability in peach is critical to allow the choice of adequate parents to confer enhanced productivity, adaptation and quality to improved varieties. With this aim we genotyped 1,580 peach accessions (including a few closely related *Prunus* species) maintained and phenotyped in five germplasm collections (four European and one Chinese) with the International Peach SNP Consortium 9K SNP peach array. The study of population structure revealed the subdivision of the panel in three main populations, one mainly made up of Occidental varieties from breeding programs (POP1OC_B_), one of Occidental landraces (POP2OC_T_) and the third of Oriental accessions (POP3OR). Analysis of linkage disequilibrium (LD) identified differential patterns of genome-wide LD blocks in each of the populations. Phenotypic data for seven monogenic traits were integrated in a genome-wide association study (GWAS). The significantly associated SNPs were always in the regions predicted by linkage analysis, forming haplotypes of markers. These diagnostic haplotypes could be used for marker-assisted selection (MAS) in modern breeding programs.

## Introduction

Since its domestication in China more than 5,000 years ago, the genetic variability of cultivated peach, *Prunus persica* (L.) Batsch, has been shaped by three major forces [1]. The first is inbreeding, favored by the self-pollinating system of peach, an unusual situation compared to most other *Prunus* species that have an efficient gametophytic self-incompatibility system. Selfing, plus the directional selection of the domestication and the selective processes of plant breeding, have resulted in a dramatic loss of variability when compared to other cultivated species of the genus [2, 3]. The second force is random drift, as a consequence of the small number of parents used in the breeding programs that started in the US in the middle of the 20th century [4, 5]. A limited number of founders may also have been used in Asian breeding programs, as inferred from the work of Li et al [6]. The third force is heterosis that, because peach is easily propagated by grafting, can be exploited in commercial breeding. The usual breeding scheme of peach and most fruit trees is the selection of individuals arising from the first generation of crosses between two accessions that are themselves, and therefore their offspring, partly heterozygous. The consequence of all this is that cultivated peach has a low level of genetic variability, high linkage disequilibrium conservation and, in most modern cultivars, a relatively high level of heterozygosity. This has been demonstrated using sets of SSR markers with good genome coverage [6,7], and, more recently, using resequencing data [8,9].

Due to its economic value (second, after apple, among temperate fruits) and its self-pollinating behavior, peach has been one of the tree species most amenable to genetic studies [10, 11], becoming a model system for genomics research in the Rosaceae. In particular, the genomes of different *Prunus* species are highly conserved [12], allowing many major genes and QTLs from peach and other *Prunus* to be positioned on a single genetic map [13], using a set of high quality and comparable linkage maps, most developed at the end of the last century [14]. Recently, the whole genome sequence of peach has been released, including the resequencing data of several peach accessions [8], opening a new era for the development of genetic studies and their application to modern breeding in this species. It has been possible to estimate the SNP variability of this species [8, 9, 15, 16] and a 9k SNP array v1 [17] has been developed by the International Peach SNP Consortium (IPSC). This SNP array is currently being used for genetic analysis [18–22].

The high abundance and relatively low cost of SNPs compared to SSRs allows for a complementary and deeper study of peach germplasm variability. In this paper, we used the IPSC 9K SNP array [17] to genotype a large collection of peach accessions including an ample set of Occidental and Oriental materials. Our results confirm previous findings on variability parameters and subpopulation structure, provide a finer analysis of linkage disequilibrium (LD), and allow the whole-genome association analysis of a set of morphological characters with Mendelian inheritance.

## Results

### IPSC 9K SNP array performance

We genotyped 1,580 *Prunus* accessions (1,576 *P*. *persica* plus four *Prunus*-related species) using the IPSC peach 9K SNP array v1 [17]. The average call rate (number of calls divided by the number of assayed SNPs) was 0.839 and, after exclusion of 40 samples with the poorest quality (call rate lower than the average call rate minus 0.1), the mean call rate increased to 0.854. The list of varieties with genotypic data is presented in S1 Table.

The 8,144 SNPs assayed were divided into five classes from A to E (see methods) depending on their performance. The SNPs classified as A (no-Call < 5% and all three possible genotypes, AA, AB, and BB), represented 53.2% of the total (4,330). After removing those with minor allele frequency (MAF) lower than 0.05 and those with poor clustering in GenomeStudio Data Analysis software (Illumina Inc.), the final set of class A SNPs was 4,271 (52.4%). The percentage of polymorphic SNPs increased to 65.5% when the SNPs in class B (null allele or preferential annealing) and C (duplicated sequences/genes) were included (Table 1). Only the 4,271 class A SNPs were used for subsequent analysis, corresponding to an average of 18.6 SNPs/Mb (considering the total peach genome size of 230 Mbp) and approximately equivalent to 8.2 SNPs/cM of the reference *Prunus* map (519 cM as in Dirlewanger et al. [12]).

**Table 1:** Number of SNPs per class of the IPSC 9K array in a collection of 1,580 *Prunus* accessions.

Class	Class description	# SNPs
A	No Call <5%, three genotypes	4,330
B	Null allele or preferential annealing	230
C	Duplicated sequences/genes	778
D	False SNPs	772
E	Failed	2,034
**Total**		8,144

### Germplasm characterization

We compared all pairs of accessions to exclude known and unknown sport mutations or synonyms from the analysis, and detected 173 groups encompassing 473 cultivars with two or more cultivars with identical genotype (more than 98% of their SNP genotypes identical). Consequently, 300 cultivars were removed, retaining only one accession for each unique genotype. The largest group of clones contained 11 genotypes, two had nine, eight, seven and six genotypes, respectively, and the rest were distributed in four groups of five, 17 of four, 28 of three and 115 of two genotypes. Most (76 out of 83) of the samples with the same name had the same genotype, including all those introduced as controls (‘Rich Lady’ and ‘Diamond Ray’ with five replicates each, and ‘Maycrest’, ‘Big Top’, ‘Elegant Lady’ and ‘Jing Yu’ with four replicates, each from a different germplasm bank). Five pairs of accessions with the same name from two different germplasm collections (‘Akatsuki’ ‘Siberian C’, ‘Suncrest’, ‘Fei Cheng Bali’ and, ‘Wu Yue Xian’), expected to be identical, were different. Additionally one replicate out of three of ‘Armking’ and one out of four of ‘Rubirich’ didn’t present the expected genotype.

Some known sports were included in the panel. In all cases they grouped together. The largest group of identical genotypes included all known sports of ‘Springcrest’: ‘Maycrest’ (four replicates, each from a different repository), ‘Early Maycrest’, ‘Queencrest’, ‘Springbelle’, ‘Springold’ and ‘Springlady’, plus two cultivars of unknown origin, ‘Harken’ and ‘San Giovanni’. Their genotypes were identical for all markers analyzed. Another group included ‘O’Henry’ and its sports ‘John Henry’ and ‘Summer Lady’. In this case, differences were identified in 11 SNPs, with ‘O’Henry’ differing from ‘John Henry’ by nine SNPs and 11 from ‘Summer Lady’, with ‘John Henry’ and ‘Summer Lady’ only differing by one SNP. All these SNPs were located at a region of 5.4 Mb at the top of chromosome 6 (between markers SNP_IGA_604703 and SNP_IGA_621593), where there was a large amount of missing data for many other SNPs in the derived sports ‘John Henry’ (41 missing data out of 92 SNPs) and ‘Summer Lady’ (38 out of 92). A similar pattern was observed for the same genomic region in ‘Redskin’ and its sport ‘Redkist’ (13 differences; 44 missing data out of 92 SNPs in ‘Red Kist’) and in the two groups of cultivars classified as having the same genotype and of unknown relationship: ‘Vittorio Emanuele’ and ‘Paola Matteucci’ (differing by 8 SNPs in the same region and with ‘Paola Matteucci’ having 60 missing data out of 92 SNPs) and four Italian accessions with different names ‘Pieri 81’, ‘Bella Lucia’, ‘Vaccaro Roccalmunto’ and ‘Zingara Nera’, where the former three were identical and the latter differed by four SNPs in this region and had 46 out of 92 SNPs with missing data. In all cases, the differences in SNPs occurred at loci that were heterozygous in the complete accessions and homozygous in those having missing data. A similar case but involving a different region was observed between cultivars ‘Springtime’ and ‘Starcrest’. These were identical in all markers except for eight SNPs located in the upper part of chromosome 4, within a region of 17.9 Mb where there was also a large amount of missing data (179 in ‘Springtime’, 231 in ‘Starcrest’, 178 of them in common with the total 581 markers in the region). As in the previous cases, all distinctive SNPs were homozygous in one variety (‘Starcrest’) and heterozygous in the other.

### Cultivar variability and population structure

A total of 1,240 unique accessions were genotyped with the 4,271 class A SNPs. The observed mean heterozygosity (H_o_) per individual was 0.286, ranging from 0.003 in ‘Burrona di Rosano’ to 0.680 in ‘Zheng huang 4’. H_o_ per SNP ranged from 0.046 in SNP_IGA_762094 to 0.781 in SNP_IGA_550718. Chromosome 6 (Ho = 0.28) was the least heterozygous while chromosome 1 had more loci in heterozygosis (Ho = 0.33). The mean expected heterozygosity (H_e_) was 0.39, ranging from 0.055 to 0.500. The mean average inbreeding coefficient (F = (H_o_—H_e_) / H_o_) was 0.27, ranging from -0.705 to 0.9922. The vast majority of the markers (98.5%) showed significant (p < 0.001) deviation from the Hardy-Weinberg Equilibrium (HWE).

**Fig 1:**
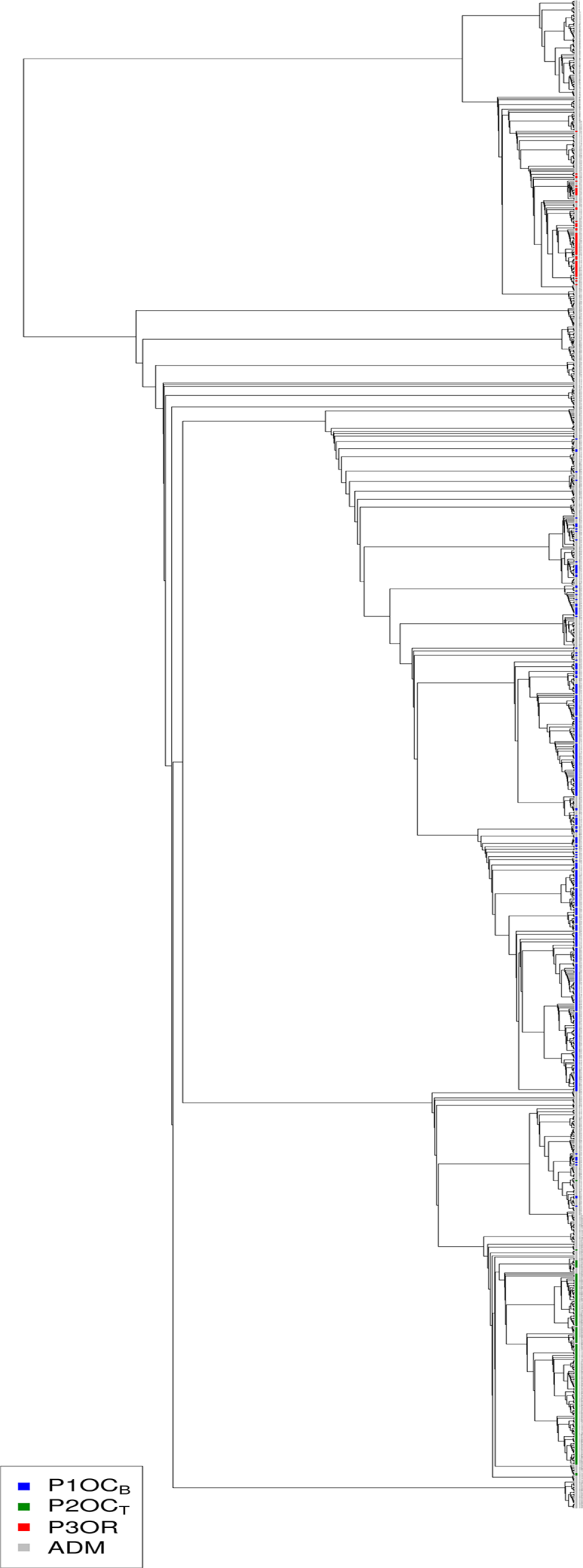
UPGMA tree for the 1,240 accessions. Colors indicate the assignment into populations obtained by STRUCTURE analysis at K = 3. Blue: Occidental/Breeding (P1OC_B_); Green: Occidental/Traditional (P2OC_T_); Red: Oriental (P3OR); Gray: Admixture (ADM). Accession names have been replaced by numbers; correspondence between number and accession is provided in S1 Table.

A phylogenetic dendrogram with the 1,240 unique accessions (Fig 1) clearly revealed two main groups, one with Oriental accessions and the other including those from Occidental origin. Two clusters could be distinguished within the Occidental group, where the traditional/non-breeding accessions were differentiated from those derived from breeding programs. The position of each accession in the dendrogram is provided in the S1 Table.

**Fig 2:**
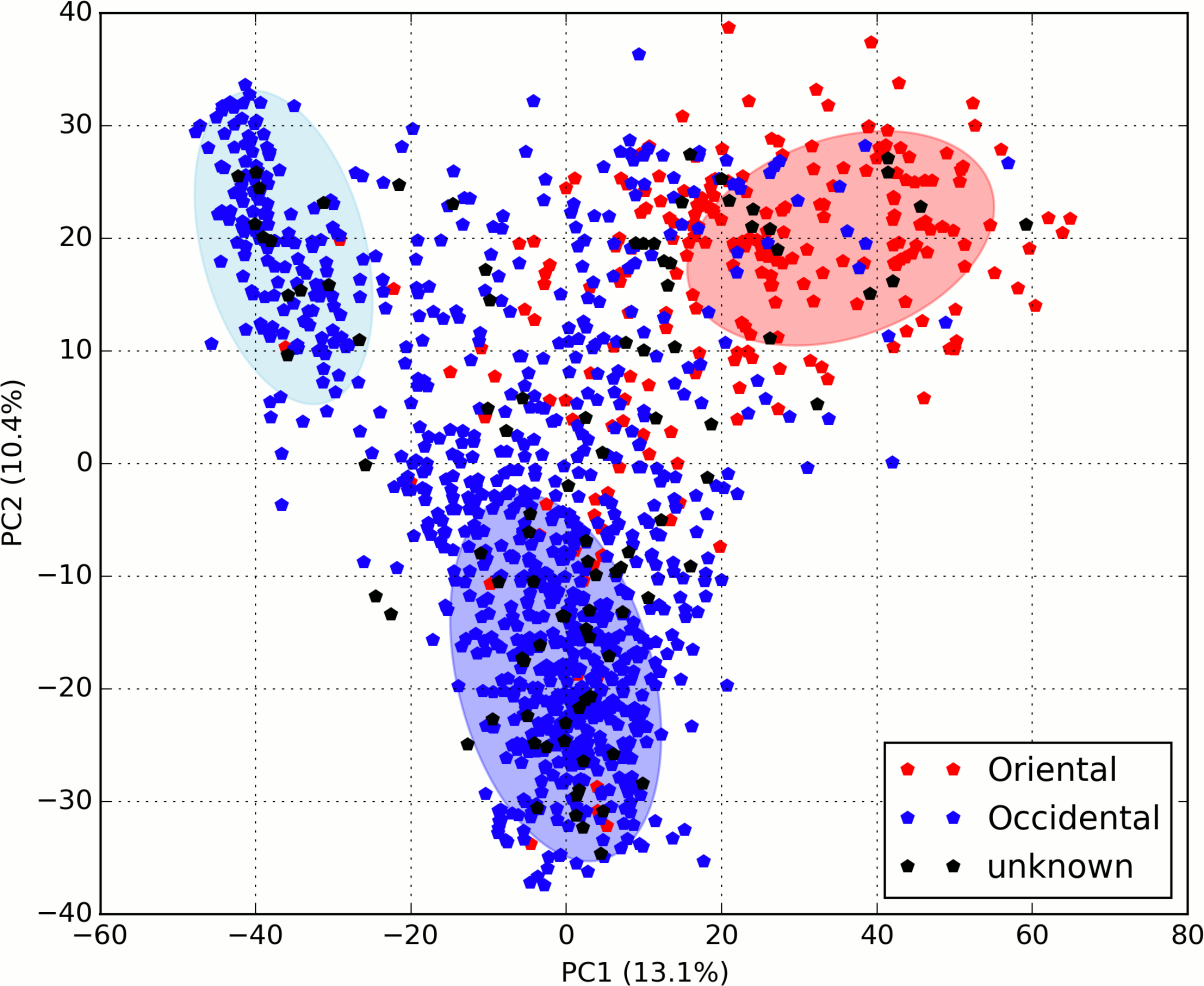
Principal component analysis of the 4,271 positively genotyped SNPs in the 1,240 unique peach accessions. Occidental and Oriental accessions are represented with blue and red colors, respectively. Black symbols indicate accessions with unknown origin.

A first approximation to the study of population structure was obtained using principal component analysis (PCA) for the complete set of SNPs, which separated the Oriental and Occidental accessions (Fig 2). The Occidental accessions were clearly separated into two major groups, one including the traditional/non-breeding accessions and the other with varieties used and obtained in modern breeding programs. The separation in three clusters was not absolute and a discrete number of accessions occupied a centric position. The majority of these accessions are known to come from crosses between accessions in different clusters.

The software STRUCTURE [23] was used to obtain a more detailed picture of the stratification in the panel. In agreement with the results of the PCA, the most probable number of subpopulations inferred by this method (K) was three. Considering only the accessions with a subpopulation membership coefficient higher than 0.8, the three subpopulations are: population 1, the Occidental materials used in modern breeding programs (P1OC_B,_ with 352 accessions), population 2, the traditional/non-breeding occidental accessions (P2OC_T,_ 165 accessions) and population 3, the oriental accessions (P3OR, 58 accessions). At K = 2, the division between the Oriental and Occidental cultivars was already evident. Increasing the number of subpopulations to four the Occidental breeding accessions were divided into two subgroups: the first comprising the vast majority of nectarines and the second the vast majority of peaches. Further increments of K maintained these four subpopulations, with additional ones empty or including only a few individuals with a membership coefficient ≥ 0.8. At all K values, more than half of the individuals (665) were not assigned to a single population and were considered as admixed (ADM) (S1 Fig). The position in the UPGMA tree of the accessions assigned into each subpopulation at K = 3 is presented in Fig 1, where accessions belonging to P1OC_B_, P2OC_T_ and P3OR are colored in blue, green and red, respectively. Gray color indicates accessions in the ADM subpopulation.

Distance between populations was calculated through the Fst statistic. Genetic differentiation between P2OC_T_ and P3OR and between P1OC_B_ and P3OR were moderate (Fst = 0.14 and Fst = 0.13, respectively), while P1OC_B_ and P2OC_T_ showed a greater differentiation (Fst = 0.18)

### Linkage disequilibrium (LD)

The LD, measured as r^2^, was calculated in the three subpopulations and in the admixed accessions separately, as well as in all 1,240 accessions together using a modified r² that corrects for population structure and relatedness [24].

**Fig 3:**
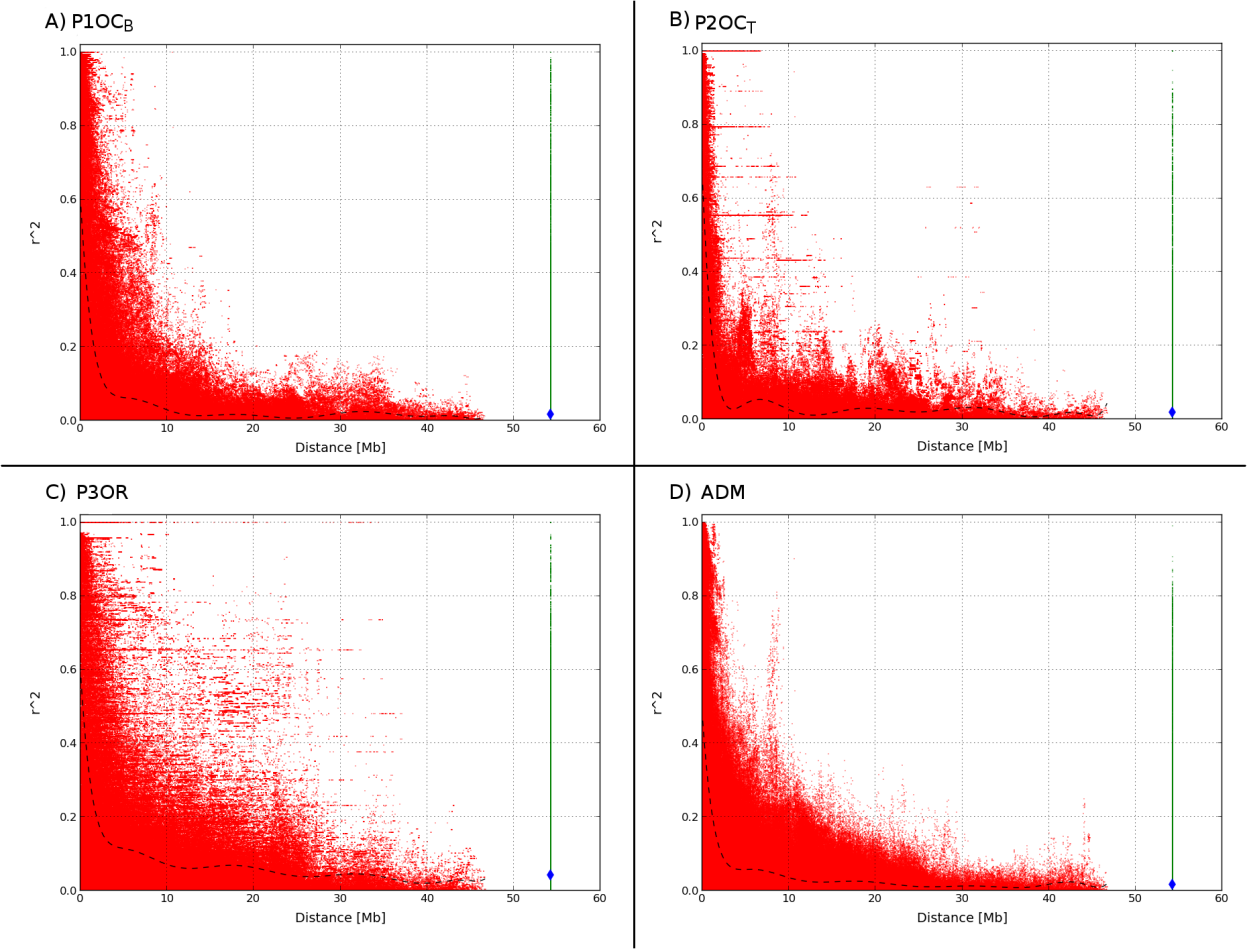
Decay of linkage disequilibrium in a collection of 1,240 peach accessions. The LD (r²) was calculated for each intra-chromosomal pair of SNPs (red) and for all the inter-chromosomal (green) pairs of SNPs. The blue dotted line indicates the polynomial fitting of the intra-chromosomal comparisons and the average inter-chromosomal comparisons.

Analyzing the SNP data in each subpopulation, 188, 949 and 353 out of the 4,271 SNPs in P1OC_B_, P2OC_T_ and P3OR, respectively, failed the frequency test (MAF < 0.01) and/or the non-calling cut-off of 5% and were discarded. The average value of intra-chromosomal r^2^ was 0.096 in P1OC_B_ and P2OC_T_, 0.133 in P3OR, and 0.082 in the admixed individuals. As expected, the average value of inter-chromosomal r^2^ was smaller than the intra-chromosomal and was 0.016 in P1OC_B_ and admixed individuals, 0.018 in P2OC_T_, and 0.042 in P3OR. The LD decayed with distance between markers in all subpopulations. The average value of r^2^ dropped below 0.2 at 1.4, 1.0, 1.8, and 0.8 Mb in P1OC_B_, P2OC_T_, P3OR and ADM, respectively (Fig 3).

Chromosomal heat-maps showed the presence of several blocks with a high level of LD spread in almost all the chromosomes, generally spanning hundreds of kilobases (S2–S6 Figs). Three of these blocks were common to all subpopulations, two at the top end of chromosome 4, of approximately 1.2 and 2 Mb, and one in chromosome 5 spanning about 2Mb. The organization of the P1OC_B_ genome in LD blocks was highest, especially chromosomes 1, 2, 3, 4 and 5. The largest block of SNPs in high LD (r^2^ >0.5) was observed in LG1, starting at 8 Mb from the top of the chromosome and spanning 12 Mb. This block was followed in length by a 6 Mb block in the same chromosome at 32 Mb from the top. In P2OC_T_ and P3OR, the SNPs at the bottom of chromosome 6, spanning 2 Mb, were in strong LD. The chromosomal heat-maps of the ADM population revealed only a few blocks of SNPs in high LD, most disappearing when the whole collection of 1,240 cultivars were analyzed with an LD statistic that corrects for population structure and relatedness, only the three common to all four subpopulations remaining.

### Genome-wide association and haplotypes for major genes

**Fig 4:**
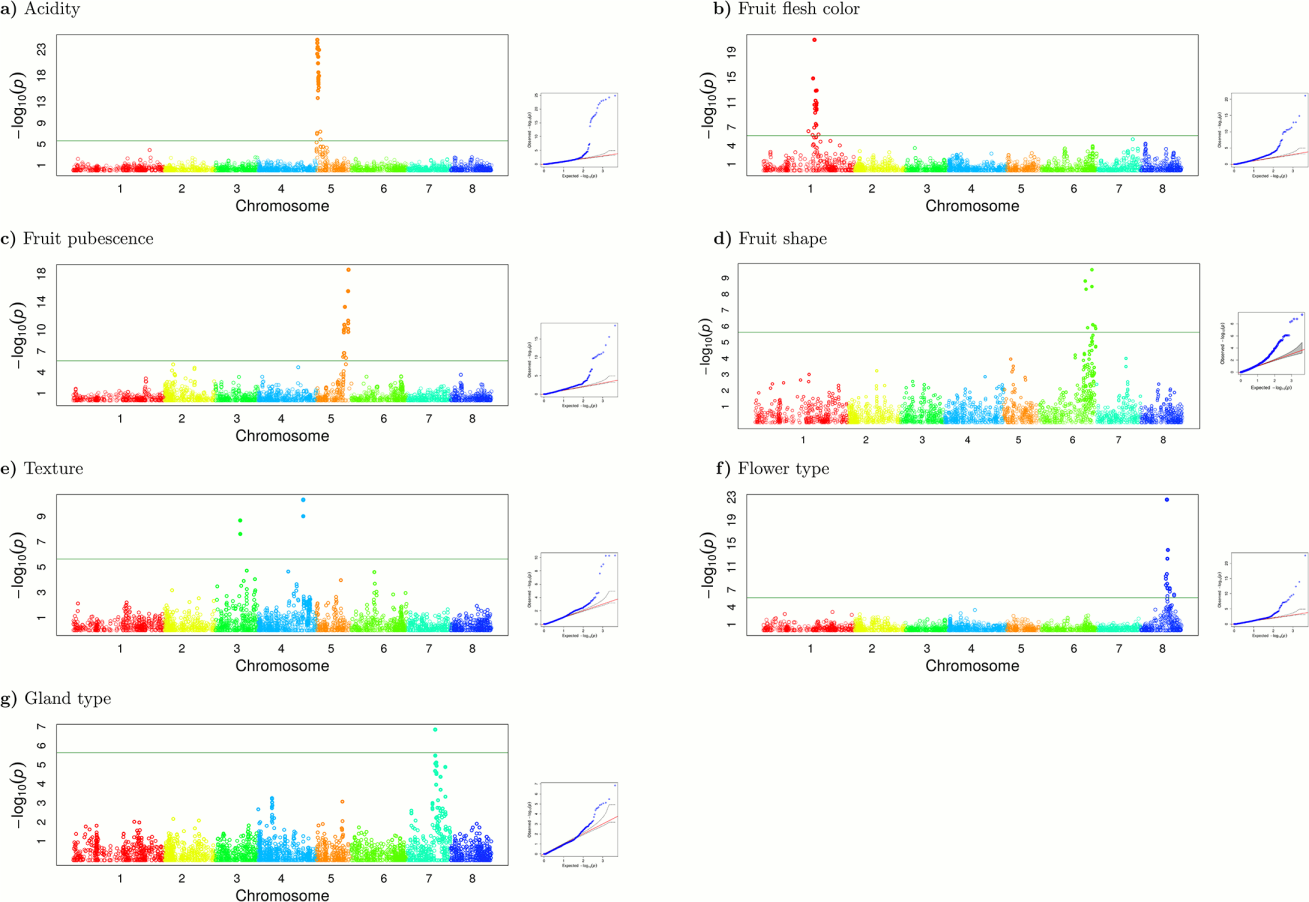
Genome wide association results for seven qualitatively inherited characters in peach. Each Manhattan plot represents one phenotypic trait. Chromosomes are marked with a different color on the horizontal axis. The horizontal green line represents the significance threshold for the association of each character.

Between 832 and 1,071 of the 1,240 genotyped plants were phenotyped for the following traits, known to be determined by single Mendelian genes [10]: fruit pubescence (*G/g*), fruit shape (*S/s*), fruit flesh color (*Y/y*), non-melting/melting flesh (*M/m*), titrable acidity (*D/d*), leaf gland type (*E/e*), and showy/non-showy flower type (*Sh/sh*) (see Methods for details). For all except fruit shape, the two phenotypic classes were homogeneously distributed. For the trait of fruit shape, the flat individuals (57) were underrepresented compared with round ones (1,003). Association between single SNPs and traits was identified, taking into account kinship and structure. The average value of kinship was 0.58, on a matrix scaled at 2, corresponding to about one quarter of the genome equivalent for example to a grandparent-grandchild, or a half sib relationship. We found significant association for all the traits (-log_10_(p) > 5.63) (Fig 4, S2 Table).

#### Titratable acidity (TA)

The acid/sub-acid fruit trait has been mapped at the beginning of LG5 [25], where the sub-acid allele behaves as dominant. A total of 405 and 427 accessions were classified as acid (TA>8.2 mEq/100 ml. [26]) and sub-acid, respectively. Twenty-three SNPs at the beginning of chromosome 5 were associated with fruit acidity (Fig 4A). The associated SNPs cover a region of about 1.8 Mb (scaffold_5: 467,067..2,270,122) in agreement with the reported position for the D locus [25]. SNP_IGA_544640 (scaffold_5: 629,641) showed the strongest association with the trait, with a p-value of 1.5E-25 and MAF of 0.21. The most frequent allele was observed in 71.4% of sub-acid varieties, in either homozygosity or heterozygosity, while this allele was only present in 3.2% of the acid varieties. The genetic variance (heritability) explained by the model adopted for the association was 0.66. In total, the 23 SNPs produced 99 haplotypes, imputed using fastPHASE [27], with an average heterozygosity of 17.13%. Four haplotypes showed a frequency higher than 5%, two being strongly associated with the sub-acid phenotype (chi-squared p-value < 2.2E-16).

#### Fruit flesh color

At least three different recessive alleles of the CCD4 gene (ppa006109; scaffold_1:25,639.445..25,641.500) are responsible for the yellow flesh in peach, one produced by a mutation in a microsatellite at the first exon of the gene, one by an SNP in the second exon and one by the insertion of a transposable element [28]. In our collection, a total of 525 accessions were yellow-fleshed (*y*) while 499 were white (*Y*). The chromosomal heat-maps showed that the SNPs flanking this gene were in strong LD in a region 2 Mbp long (S1–S5 Figs). This block was shorter in P2OC_T_ and disappeared in P3OR (where most of the cultivars had white flesh) and ADM. Association analysis identified 21 SNPs located in a 5.2 Mb region in linkage group G1 (scaffold_1:23,352,245..28,551,537) associated to this trait (Fig 4B). The SNP with the strongest association was SNP_IGA_90213 (scaffold_1: 26,479,525), with a p-value of 8.03E-22 and MAF of 0.21. The most frequent variant at this position was strongly linked to white flesh, being present in 61.9% of the white and 6.8% of the yellow varieties. The heritability explained by the allele was 0.65. The 21 associated SNPs were distributed in 152 imputed haplotypes. Six of them showed a frequency higher than 5%, all associated with the trait (Chi-square p-value <0.01), and an average heterozygosity of 92%.

#### Fruit hairiness

The recessive glabrous fruit trait (nectarine) has recently been associated to a retrotransposon insertion in an MYB gene (ppa026143; scaffold_5:15,897,836..15,899,002) [29]. Of the 1,071 accessions studied, 792 were hairy (peaches, carrying the *G* allele) and 290 glabrous (nectarines, homozygous for the *y* allele). We found 19 SNPs associated with the trait, mapping in G5 (scaffold_5: 13,952,707..16,774,236). The average MAF of the associated SNPs was 0.31 and the heritability 0.73 (Fig 4C). SNP_IGA_603047 (scaffold_5:16,774,236) showed the highest association (p-value = 2.5E-19, MAF = 0.37 and R² = 0.66). The most frequent allele was equally present in peaches and nectarines while the less frequent was almost exclusively in peach (98.2% of the varieties with this allele were peaches). PHASE imputed 123 haplotypes using the 19 associated SNPs, five with a frequency > 5% and an average heterozygosity of 8.15%. The most frequent was in high association with the recessive trait: 98% of the accessions with this haplotype in homozygosis had the recessive phenotype.

#### Flat fruit shape

Flat fruit shape is caused by a dominant allele in obligated heterozygosity, mapped at the end of LG6 [25, 30]. In our panel, only 57 cultivars were flat whereas 1,003 were round. The low number of flat accessions hampered the analysis and it was not possible to identify significant association using the full dataset. This situation could be partially explained considering the relatedness of some flat individuals, for example those from the series UFO (12.3% of flat varieties), and the strong structuring of the trait. In order to solve these drawbacks, we studied 15 independent subsets containing the 57 flat and 100 randomly chosen round cultivars from P1OC_B_ and admixed accessions. In all the subsets, a single peak of association at the end of chromosome six was evident (scaffold_6:23,101,004..26,601,733). The number of associated SNPs varied between subsets, ranging from five to 22 (Fig 4D). Five were associated with the trait in all runs (SNP_IGA_683904, SNP_IGA_684085, SNP_IGA_685825, SNP_IGA_696241, SNP_IGA_696280), with an average MAF of 0.29 and heritability of 1.0. The first two were in complete LD and showed, on average, higher association with the flat trait. Most of the flat varieties (87.7%) were heterozygous at these two loci while this percentage decreased to 8% in the round varieties, consistent with the genetics of the trait.

#### Texture

An endoPG gene, mapping at the end of LG4, has been reported to control melting flesh (M) in peach [31]. In our germplasm collection, 213 accessions were classified as non-melting and 746 as melting. We found two SNPs significantly associated in LG3 12 kb apart (scaffold_3:12,836,182..12,878,608) and four in LG4 covering a region of 11.6 kb (scaffold_4:23,497,381..23,508,950) (Fig 4E). The two SNPs associated in LG3 (SNP_IGA_341962; p-value 2.1E-9, and snp_3_12878608; p-value 2.4E-8) were in high LD (r^2^ = 0.909) and the association was due to an excess of the most frequent allele in homozygosis in the melting varieties. All four SNPs associated in LG4 (SNP_IGA_477941, SNP_IGA_477945, SNP_IGA_477951, SNP_IGA_478039) were in high LD, the first two being in complete LD (r^2^ = 1) (SNP_IGA_477941 and SNP_IGA_477945; p-value = 5.1E-11). The association was due to an excess of the less frequent allele in homozygosis in the non-melting fruits. Four and six haplotypes were imputed using the two and four associated SNPs of chromosomes 3 and 4, respectively. Two haplotypes had a frequency higher than 5% in both chromosomes. The average heterozygosity was 10.6% in chromosome 3 and 18.9% in chromosome 4.

#### Flower type (showy/non-showy)

The showy/non-showy flower locus (*Sh*) maps in LG8 [32], with the non-showy allele being dominant. Two-hundred and sixty of the accessions analyzed had non-showy flowers while 681 had showy flowers. We found 18 SNPs associated with the trait in a region of 4.3 Mb of chromosome eight (scaffold_8: 13,204,775..17,476,655) (Fig 4F). The average MAF of the associated SNPs was 0.31 and the heritability was 0.62. The most highly associated SNP was SNP_IGA_864149 (scaffold_8: 13,756,987) with a p-value of 2.73E-23, MAF of 0.36 and an r² of the model with the SNP of 0.42. The homozygous genotype of the most frequent allele of this SNP was more frequent in the showy (66.2%) than in the non-showy (10%) varieties. A total of 162 haplotypes were imputed using the 18 SNPs associated, six with a frequency higher than 5%.

#### Leaf gland type

The *E* locus, determining the globular or reniform shape of the leaf glands, maps in LG7 [33]. The globular leaf gland phenotype was present in 190 accessions and the reniform in 750. A single SNP (SNP_IGA_776161, scaffold_7:14,870,521) was associated to the leaf gland type (Fig 4G). The associated SNP had an MAF = 0.39, R² = 0.10 and heritability of 0.31.

## Discussion

Analysis of 8,144 SNPs, represented in the IPSC 9k chip, in a diverse sample of 1,580 peach accessions revealed polymorphisms in 5,378 SNPs (66%), while 727 (9%) were false (monomorphic) and 2,037 (25%) failed. These data confirm the accuracy of the validation by Verde et al. [17] using a subset of 96 SNPs of this array, where they found similar proportions (57%, 19% and 24%, respectively). Only 4,271 of the polymorphic SNPs were retained after exclusion of 1,107 that were suspected to contain null/preferential amplification alleles, correspond to duplicate sequences or had MAF <0.05.

Such an array of polymorphic markers allows comparison of genotypes at the whole-genome level, identifying features not possible to discern with the low density marker analysis assays used to date [6, 34]. One of them concerns the differences observed between some of the known and inferred groups of sports analyzed. In four cases, we observed that SNP differences among sports were concentrated at one 5.3 Mb region in the distal end of chromosome 6. These differences involved changes of marker genotype (in 1–11 SNPs) and missing data in many of the 92 markers included in our SNP array in this region (between 38 and 60 SNPs with no data). The pair of cultivars, ‘Springtime’ and ‘Starcrest’, presented a similar pattern, although in this case affecting the upper part of chromosome 4. The same region of chromosome 6 is involved in a reciprocal translocation with chromosome 8 in the red-leaved rootstocks ‘Nemared’ [35], ‘Akame’ [36] and ‘Rubira’ [37]. Our results suggest that this region of the genome may be particularly unstable, with major rearrangements occurring frequently. They also suggest that the phenotypic differences between an original cultivar and its sports may be caused by genes located in these unstable DNA fragments.

The UPGMA dendrogram separated Oriental accessions from those cultivated in Occidental countries. Breeding accessions were also clearly separated from those cultivated locally. The kinship analysis revealed that accession pairs were related on average at the level of grandparent-grandchild or half-sibs. This was in agreement with the results from PCA and the STRUCTURE software, identifying three subpopulations within the peach germplasm analyzed: P1OC_B_, containing most accessions from modern breeding programs, essentially the gene pool derived from that used by early US breeders; P2OC_T_, including the old European accessions, most of them non-melting peaches that were seed-propagated for a long time and as a consequence highly homozygous; and P3OR that encompasses the majority of Oriental accessions. Previous studies with SSRs [6, 34, 38] also showed that *P*. *persica* has a strong population stratification and identified the same groups. A recent study [9] identified a different stratification in a panel of 84 wild and cultivated peach accessions: i) wild relatives (species), ii) edible and iii) ornamental accessions. However, only a few Western and breeding accessions were included in the panel. The subpopulations detected are in general consistent with the known history of peach domestication and dispersal, where P3OR represents the original Chinese gene pool from the domestication process, and the old European materials (P2OC_T_) are those derived from the migration of the Chinese accessions throughout central Asia towards Western Europe. Some of these materials were taken to America by European settlers and were the founders, with a few accessions coming directly from China, of the US breeding programs that started in the early 20th century using the concepts of modern genetics. These programs produced a new wave of successful cultivars that are currently the basis of European and North-American peach production, constituting subpopulation P1OC_B_.

The population structure identified in this study is also consistent with the genetic bottlenecks described by Verde et al. [8] using a set of whole genome SNPs in a panel of 12 Occidental and Oriental accessions. The first bottleneck clearly denotes the domestication event and is related to the P3OR subpopulation. The second bottleneck is related to peach dissemination in the West and to the modern breeding activities that began in the US and Europe in the last century. This bottleneck is represented by the P2OC_T_ and P1OC_B_ subpopulations. In their study Cao et al. [9] suggested the existence of a single bottleneck related to the domestication event failing in identifying dissemination and breeding bottlenecks. However, they included only a few Occidental and breeding accessions in their panel; this lack likely hampered the detection of Western dissemination and modern breeding bottlenecks. A subdivision within this population that is also evident in the SSR studies [7, 34] separates peaches from nectarines, which emphasizes the fact that breeding of these two fruit types has often been separate. A large fraction of the accessions analyzed (54%) were admixed between the subpopulations identified, suggesting that breeding programs also look for useful variability crossing the boundaries of each subpopulation.

The mean observed heterozygosity was H_o_ = 0.28, a lower value than that usually reported with SSRs: H_o_ = 0.34 in a collection of Occidental cultivars [34] and H_o_ = 0.47 for a larger collection of Oriental and Occidental materials [6]. This was expected considering the higher numbers of alleles per locus of SSRs. The proportion of markers with MAF< 0.2 was low when considering all the genotypes studied in the admixed group (17.3–20.0%), and much higher for the three subpopulations (46.8% for P1OC_B_, 54.2 for P2OC_T_ and 61.3% for P3OR). This situation can be explained considering that the SNPs were identified in resequencing data of about 50 peach accessions, mostly from the P1OC_B_ and the admixed groups [17], and by the larger size of these groups (352 and 665 accessions, respectively) compared to the other subpopulations.

Population structure affects the LD throughout the genome. LD decayed with distance in all subpopulations, being faster in the ADM subpopulation. Accessions in this population contain variability from genetically distinct founding populations with different allele frequencies: thereafter they should retain the LD of the parental populations. Additionally the amount of admixture-generated LD between any two loci is highly dependent on the proportion of each population in the admixture process [39]. Faster decay of LD in ADM could be due to the remaining subpopulation stratification in P1OC_B_, P2OC_T_ and P3OR or to a relatively low participation of these three populations in the admixture process (see S1 Table).

Previous studies comparing LD dynamics have detected faster decay in Oriental compared to Occidental germplasm [6]. One reason this was not observed in our sample could be the origin of the accessions of the population; here P3OR only included mostly Chinese breed varieties, and no wild germplasm or landraces. This indicates that breeding efforts in China and Occidental countries have resulted in similar LD patterns and, according to Xie et al.[40], in a similar reduction of variability. As expected, the LD pattern was not homogeneous across the chromosome, containing areas with extensive linkage disequilibrium. These areas may correspond to recombination cold-spots and so should be observed in all populations as well as in linkage maps. Two LD blocks in LG4, one at the top and one at the bottom, and one in LG5, were observed in all populations. These regions were also observed by Verde et al.[8], who also plotted the relation between genetic and physical distance in peach chromosomes. A low ratio between genetic and physical distance, indicating a low recombination rate, was only observed for the block at the end of LG4. Selection may explain the LD blocks of the other two regions. Artificial selection has a strong effect on LD. Mosaics of large LD blocks have been found in regions carrying agronomic-related genes [41]. Another example of LD generated by selection detected in our study is the long LD block around the *Y* gene in P1OC_B,_ with most of the varieties having the recessive yellow flesh phenotype. This disappears in P3OR, where most varieties had white flesh.

Association mapping exploits natural diversity to search for functional variation. This method presents several advantages over linkage mapping but usually they are used together to validate or discard spurious associations. Here we applied association mapping to validate its use in peach. For some of the traits evaluated the genes have been cloned (as is the case of the *Y*, *G* and *F* loci) or mapped in a short interval. In all cases, the SNPs found to be associated with the traits were located in the known region. For the known genes ppa006109 for the *Y* locus, ppa023143 for *G* and ppa006839 for *F*, the most associated SNPs were 838, 875 and 849 Kb upstream of the 5’-UTR of the gene, which corresponds approximately to 1.9 cM (considering the *Prunus* genome of 519 cM and 230 Mb).

We found SNPs tightly linked to the traits considered, but their use in marker assisted selection (MAS) is not straightforward and still needs to be validated. For most of the traits we found haplotypes that explain a large fraction of the phenotypes, however the association is not complete. For example three causal alleles have been reported for the yellow flesh trait [28]. All three are loss-of-function alleles from different mutation events and, consequently, have different SNPs (or haplotypes of SNPs) associated to each allele. Here we showed the considerable extent of LD in peach and that, in populations, SNPs are structured into haplotypes which may be better at predicting the phenotype than single SNPs. Our results do strongly suggest that the use of haplotypes is more powerful than single tightly-linked SNPs in MAS. However, in most cases, these haplotypes will be population-dependent, i.e. they have to be chosen based on the frequency of the haplotypes in the parental lines used in the breeding program.

## Conclusions

A collection of 1,580 Occidental and Oriental peach accessions was analyzed with an SNP array covering the whole peach genome. Results showed that the levels and organization of variability are consistent with the known major events in the history of this species after domestication, and generally agree with published information obtained with SSR markers and SNPs.

Given the high levels of LD conservation of the peach genome, whole-genome association analysis with a relatively small number of loci (4,271) showed that SNPs significantly associated with seven major genes were always in the regions predicted by classical linkage analysis. For the genes still not cloned, our data provide sets of tightly linked markers that can be the starting point to identify candidate genes and eventually used for their cloning and molecular characterization. The results also suggest that similar analysis with characters of complex inheritance and high value, such as those related with fruit organoleptic quality and extended post harvest life, will be able to identify genomic regions containing the most important genes responsible for variation of the traits.

The information has other applications for peach breeding and germplasm management, as many commercial cultivars from private and public breeding programs have been characterized genetically to an unprecedented level of detail, enabling the study of the variability within each program and comparison of the variability between breeding programs on a whole-genome basis. This information is useful for parent selection and to search for variability at specific genomic regions not present in the set of parents used in a specific breeding program, but present in the global peach gene pool. Moreover, our results reveal that, for certain characters, the MAS strategy not only requires one or several markers sufficiently close to the gene of interest, but also identification of the different haplotypes for various markers in the region, selecting those that are diagnostic for the character based on the haplotypes of the set of parents used by each breeding program.

## Materials and Methods

### Plant materials and phenotyping

A set of 1,580 accessions of *Prunus* were selected to be representative of five germplasm collections in four different countries. Of these accessions 1576 were *P*. *persica* and four were of other related *Prunus*. In detail, 420 accessions were from the peach germplasm collection of Centro di Ricerca per la Frutticoltura (CRA-FRU, Roma), 112 and 239 were provided by the National Institute of Agronomic Research (INRA) of Avignon (France) and Bordeaux (maintained in the Prunus Genetic Resources Center, France), respectively, 367 were from the peach germplasm collection of IRTA at the experimental stations of La Tallada d’Empordà and Gimenells (Spain), 160 were obtained from the collection of University of Milan (Italy), and 282 from the peach collection of Zhejiang University (Hangzhou, China). The complete list of the accessions is reported in S1 Table.

Between 50 and 100 mg of young leaves from each accession were freeze-dried and used for genomic DNA extraction with the DNeasy 96 Plant Mini Kit (Qiagen, Valencia, CA, USA) following the manufacturer’s protocol.

The phenotype of a variable number of plants was available for seven monogenic traits: 1,071 for fruit pubescence (peach/nectarine), 1,060 for fruit shape (round/flat), 1,024 for fruit flesh color (yellow/white), 959 for fruit texture (melting/non-melting), 832 fruit acidity (acid/sub-acid), 940 for gland type (round/reniform), and 741 for flower type (showy/non-showy). All the traits were coded as qualitative.

### SNP genotyping

1,580 *Prunus* accessions were genotyped using the International Peach SNP Consortium (IPSC) peach 9K SNP array v1 [17]. SNP genotypes were scored with the Genotyping Module of the GenomeStudio Data Analysis software (Illumina, Inc.) using the default parameters.

The SNPs were divided into five categories: A, B, C, D, and E. The first three included the polymorphic SNPs and the last two the non-polymorphic. The SNPs with GenTrain higher than 0.4 and GeneCall 10% higher than 0.2 and at least two genotypic classes were classified as polymorphic. The three classes of polymorphic SNPs were defined as follows:

A. SNPs with less than 5% of No Call (failed genotyping) and all three possible genotypes (AA, AB, BB) defined.
B. SNPs with a potential null allele or preferential annealing. Between 5 and 50% of individuals showed a normalized signal intensity value R < 0.2 and the remaining individuals represented at least two of the three genotypic classes.
C. Probable presence of duplicated sequences/genes. These SNPs were characterized by the absence of one homozygous cluster with an over-representation of heterozygous individuals, and a percentage of No Call <5%.

The non-polymorphic SNPs were divided as:

D. False SNPs. Characterized by a single genotypic class and a percentage of No Call < 5%.
E. Failed SNPs. Having more than the 50% of No Call and/or with a GenTrain < 0.4 and/or a GeneCall 10% < 0.2.

All further analysis were performed using all Class A SNPs with a minor allele frequency (MAF) higher than 0.05. Genotypic data have been uploaded to GDR database (http://www.rosaceae.org/) under the accession number tfGDR1013 and in the FruitBreedomics database (http://bioinformatics.tecnoparco.org/fruitbreedomics/).

### Phylogenetic tree

The distance matrix was calculated using TASSEL [42], as 1—IBS (identity by state) similarity, being IBS the probability that alleles drawn at random from two individuals at the same locus are the same. For clustering, the distance of an individual from itself was set to 0. The clustering was performed using the R package hclust with the UPGMA method.

### Population Structure and Fst

PCA analysis was performed using the “prcomp” R package, freely available at http://stat.ethz.ch/R-manual/R-patched/library/stats/html/prcomp.html, after genotype numericalization as follows: The following approach was followed for numericalization: the higher value (2) was assigned to the heterozygous state, the lower value (0) was assigned to the less frequent homozygous state, and the intermediate state (1) was assigned to the most frequent homozygous state.

Population structure was studied with the “prcomp” function of R and with the Structure v.2 [23] software. This program uses a clustering method that identifies K subgroups of individuals with distinctive allele frequencies. Individuals can be members of multiple subpopulations with a different coefficient, with the sum of all being equal to 1. To check for population stratification, the program was run under the admixture model assumption with correlated allele frequencies. The run used 100,000 interactions after a burn-in of 10,000 for a value of K ranging from 2 to 20. To avoid bias due to tightly linked markers and to speed up the computation time, the SNPs were pruned based on LD using PLINK [43] to give a final 1,506 SNPs. Pruning was using the “indep” function with a window size of 50 SNPs, five SNPs shift of the window at each step, and a variance inflation factor (VIF) threshold of 10 (equivalent to prune to an LD of 0.75). The VIF is 1/(1-R^2^) where R^2^ is the multiple correlation coefficient for an SNP being regressed on all other SNPs simultaneously. The most probable number of populations was estimated using the method proposed by [44]. The fixation index (Fst) was calculated as in [45] using a custom python script.

### HWE and MAF

The Hardy Weinberg equilibrium and the minor allele frequency were calculated for each SNP using PLINK [43]. The SNPs showing severe distortion of the HWE (p<10e-4) and/or MAF lower than 0.05 were discarded from further analyses.

### Linkage disequilibrium

Given that the phases between alleles at two heterozygous loci are unknown, the linkage disequilibrium (LD) was calculated using the squared correlation based on genotypic allele counts as implemented in PLINK [43]. Additionally, the LD was calculated using the R-package LDcorSV [24], able to correct the classical r^2^ measure for population structure and relatedness. To measure LD, SNPs with a minor allele frequency (MAF) lower than 5% were discarded.

The curve representing the observed r^2^ values was computed using a polynomial fit as implemented in the “numpy” module (http://docs.scipy.org/doc/) of python v.2.7.3.

### Kinship

The kinship matrix was computed using the VanRaden algorithm [46] as implemented in the GAPIT R package [47].

### Genome wide associations

Association analysis was using the compressed mixed linear model [48] implemented in the GAPIT R package [47]. Association tests were run using two approaches. One was a global approach where an MLM model for all individuals was run using population structure (k coefficients of ancestry, Q), and a genetic covariance matrix (kinship matrix, K) used as cofactor for SNP effects. The other was a population level approach where each sampling population was run in a separate analysis using the MLM as outlined above. Significant associations were determined using both a B–Y FDR P-value adjusted for multiple testing [49] and a Bonferroni adjustment at the α = 0.01 level. Prior to the analyses, SNP markers were filtered for MAF of ≥ 0.05, and a minimum genotyping success of 90%.

The SNPs associated to each trait were phased independently using fastPHASE [27]. For each run of fastPhase 20 random starts and 100 iteration of the EM algorithm were used. To test the association of the inferred haplotypes with the analyzed phenotypes a chi-squared test was performed for each haplotype. The null hypothesis was that the number of accessions showing the two alternative phenotypes was the equal.

## Supporting Information

S1 Fig STRUCTURE output for 1,240 peach accessions with K varying from 2 to 7. (TIFF)

S2 Fig Heat map representing the chromosomal level of LD measured as r² in P1OC_B_ (Occidental accessions). The diagonal black line indicates the physical position of the SNPs on the chromosome.
(TIFF)

S3 Fig Heat map representing the chromosomal level of LD measured as r² in P2OC_T_ (Occidental traditional accessions). The diagonal black line indicates the physical position of the SNPs on the chromosome.
(TIFF)

S4 Fig Heat map representing the chromosomal level of LD measured as r² in P3OR (Oriental accessions). The diagonal black line indicates the physical position of the SNPs on the chromosome.
(TIFF)

S5 Fig Heat map representing the chromosomal level of LD measured as r² in the admixed individuals. The diagonal black line indicates the physical position of the SNPs on the chromosome.
(TIFF)

S6 Fig Heat map that represents the linkage disequilibrium calculated as r² corrected for population structure and relatedness. The diagonal black line indicates the physical position of the SNPs on the chromosome.
(TIFF)

S1 Table List of varieties analyzed. (1) Asterisks indicate accessions from two different germplasm collections with the same name but with different genotype. (2) n/p = nectarine/peach; y/w = yellow/white flesh; f/nf = flat/non flat fruit shape; f/c/s = freestone/clingstone/semi-clingstone; m/n = melting/non melting; s/a = sub-acid/acid; r/g = reniform/globose leaf gland; s/ns = showy/non-showy flowers. (3) Numbers indicate the position of the accession in the Fig 1 phylogenetic tree, starting from the top branch.
(XLSX)

S2 Table SNPs associated with each of the traits studied. (XLSX)
