# Supplementary figures and images for: Whole-Genome Analysis of Diversity and SNP-Major Gene Association in Peach Germplasm
Source: PLoS One. 2015 Sep 9;10(9):e0136803. doi: 10.1371/journal.pone.0136803 (PMC4564248; doi:10.1371/journal.pone.0136803)

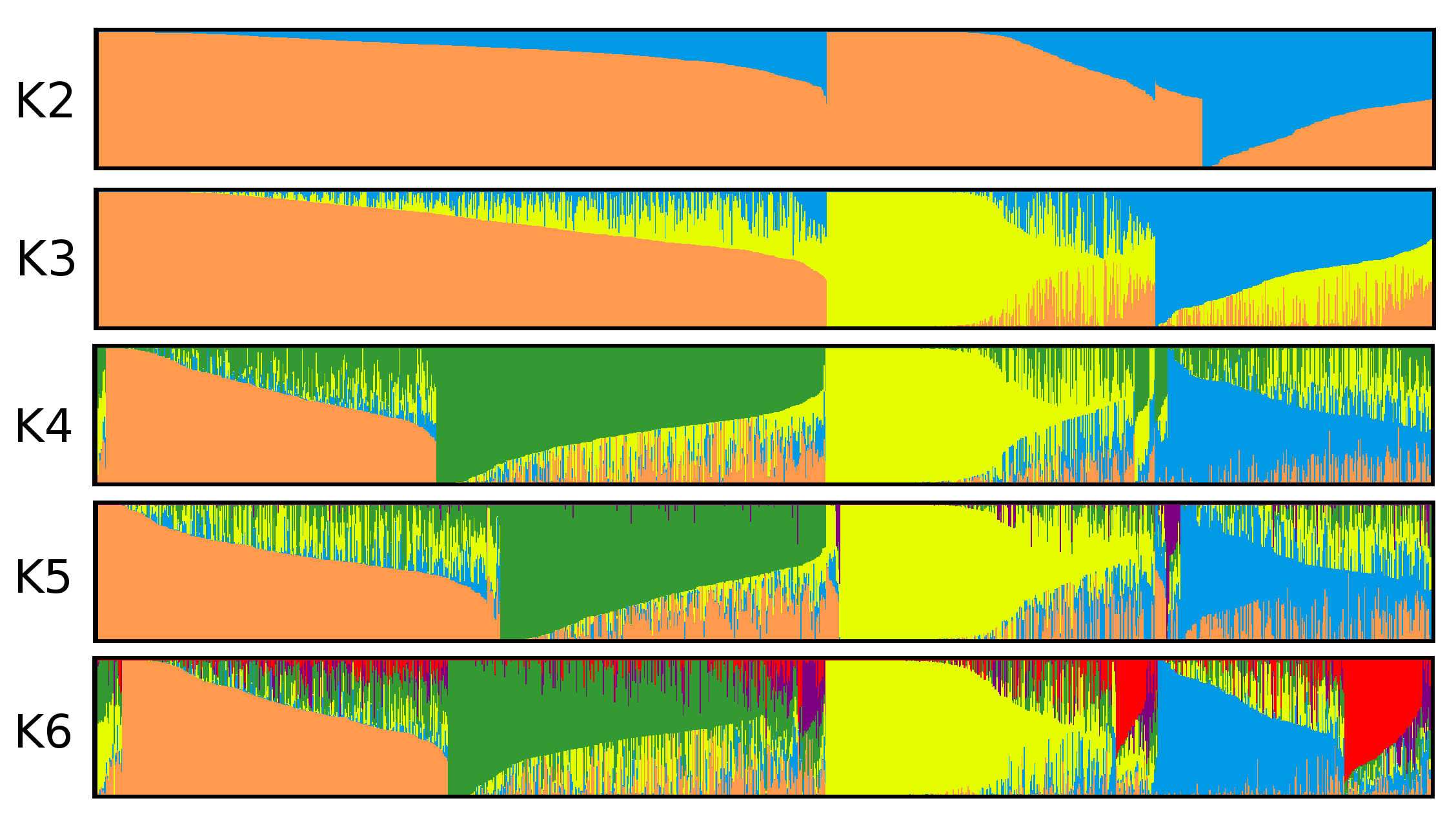

Supplement: S1 Fig — (TIFF) [file pone.0136803.s001.tiff]

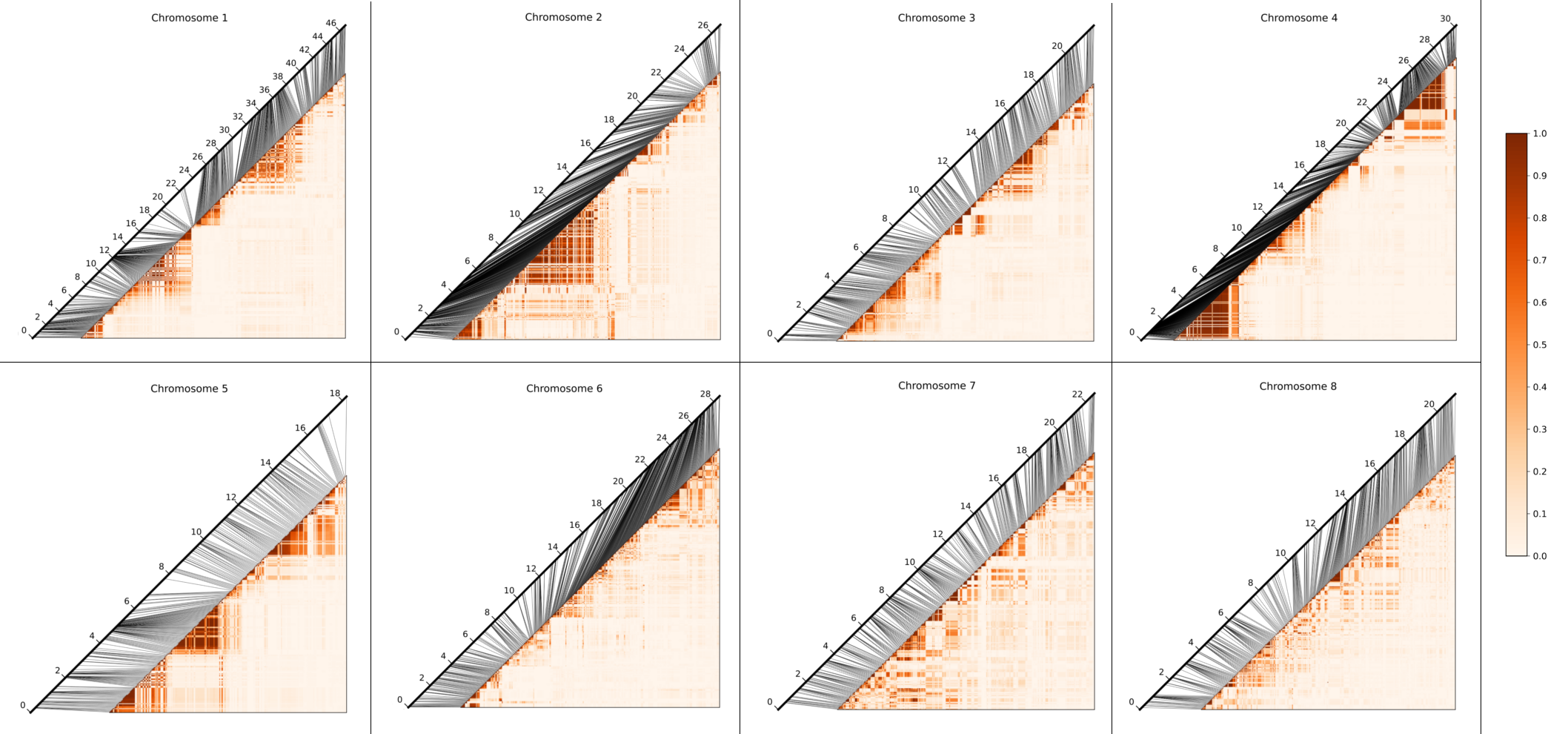

Supplement: S2 Fig — The diagonal black line indicates the physical position of the SNPs on the chromosome. (TIFF) [file pone.0136803.s002.tiff]

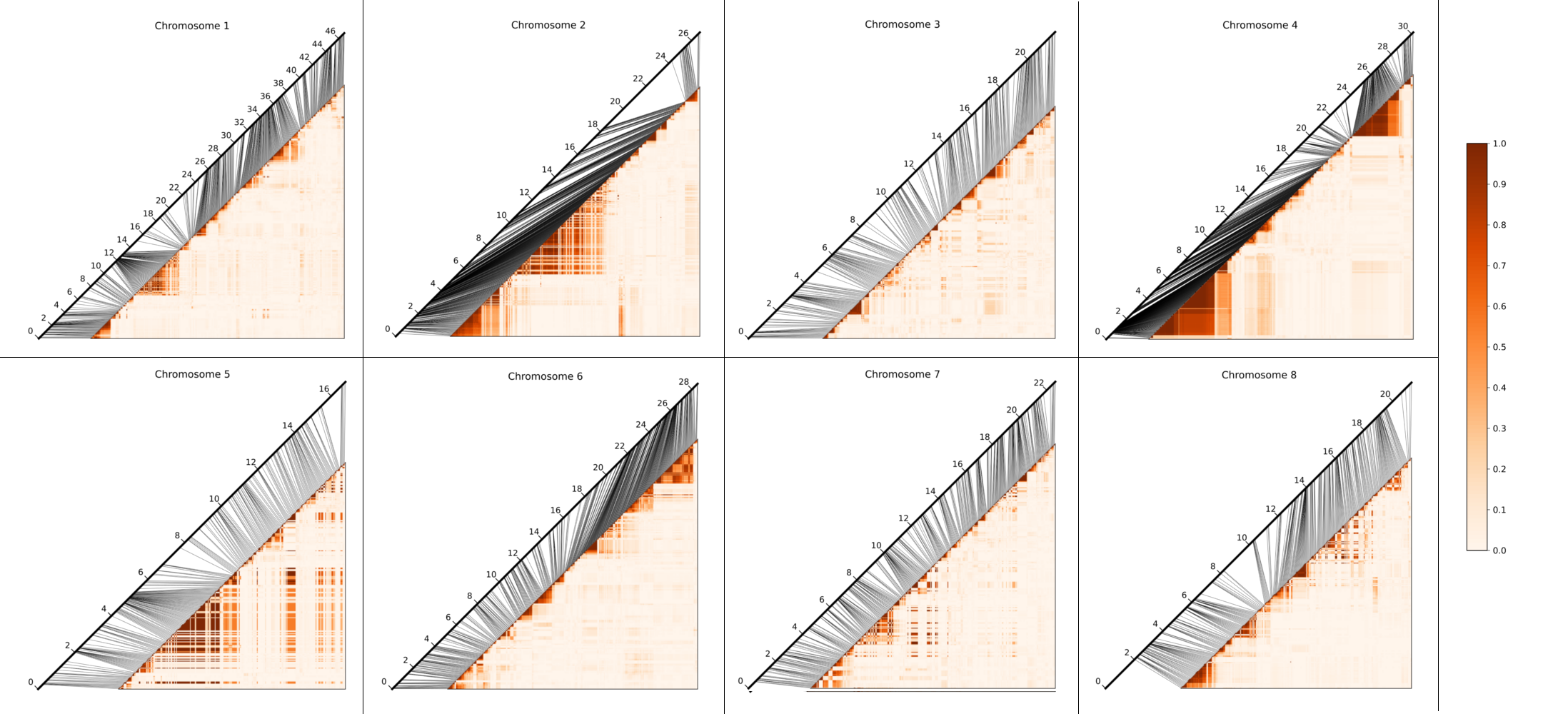

Supplement: S3 Fig — The diagonal black line indicates the physical position of the SNPs on the chromosome. (TIFF) [file pone.0136803.s003.tiff]

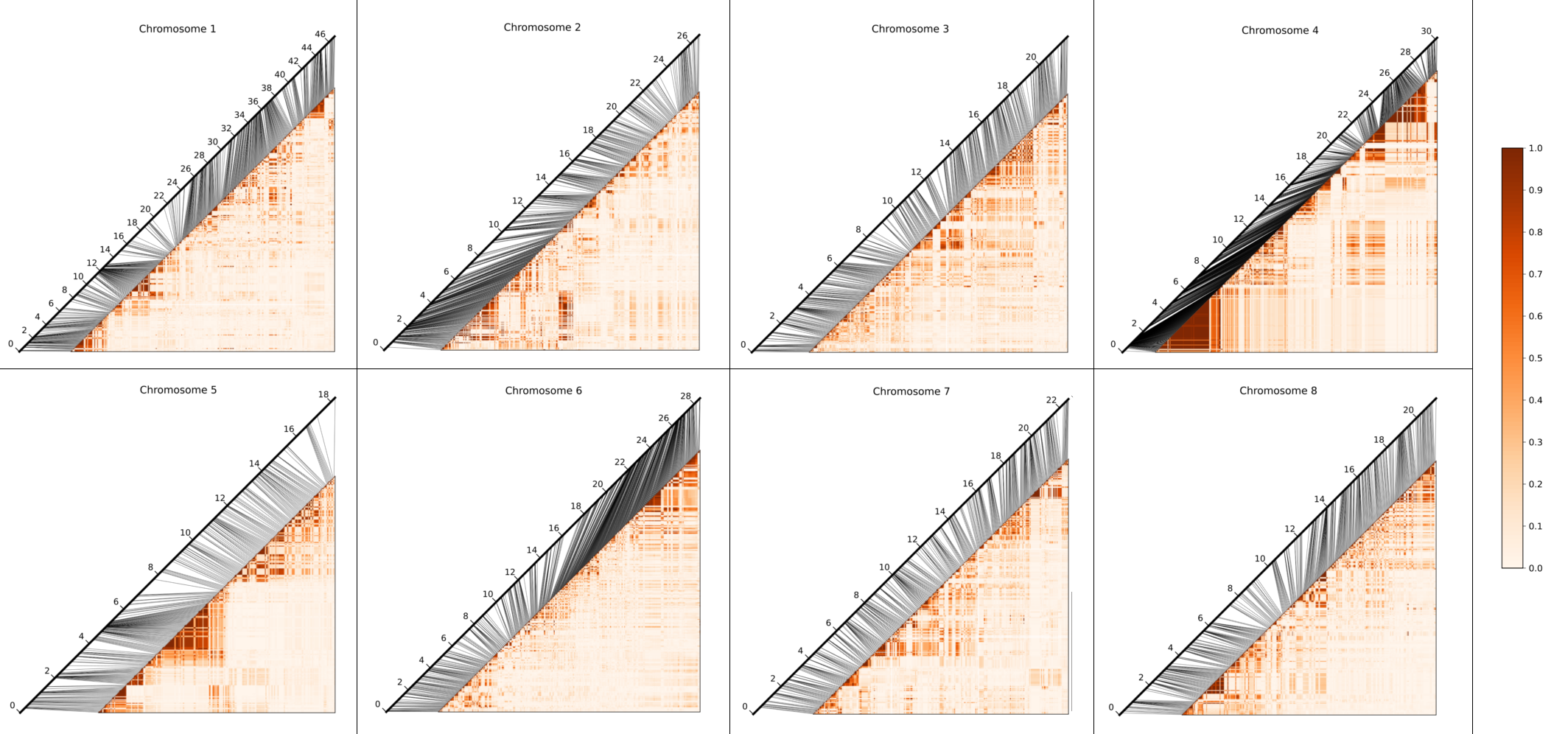

Supplement: S4 Fig — The diagonal black line indicates the physical position of the SNPs on the chromosome. (TIFF) [file pone.0136803.s004.tiff]

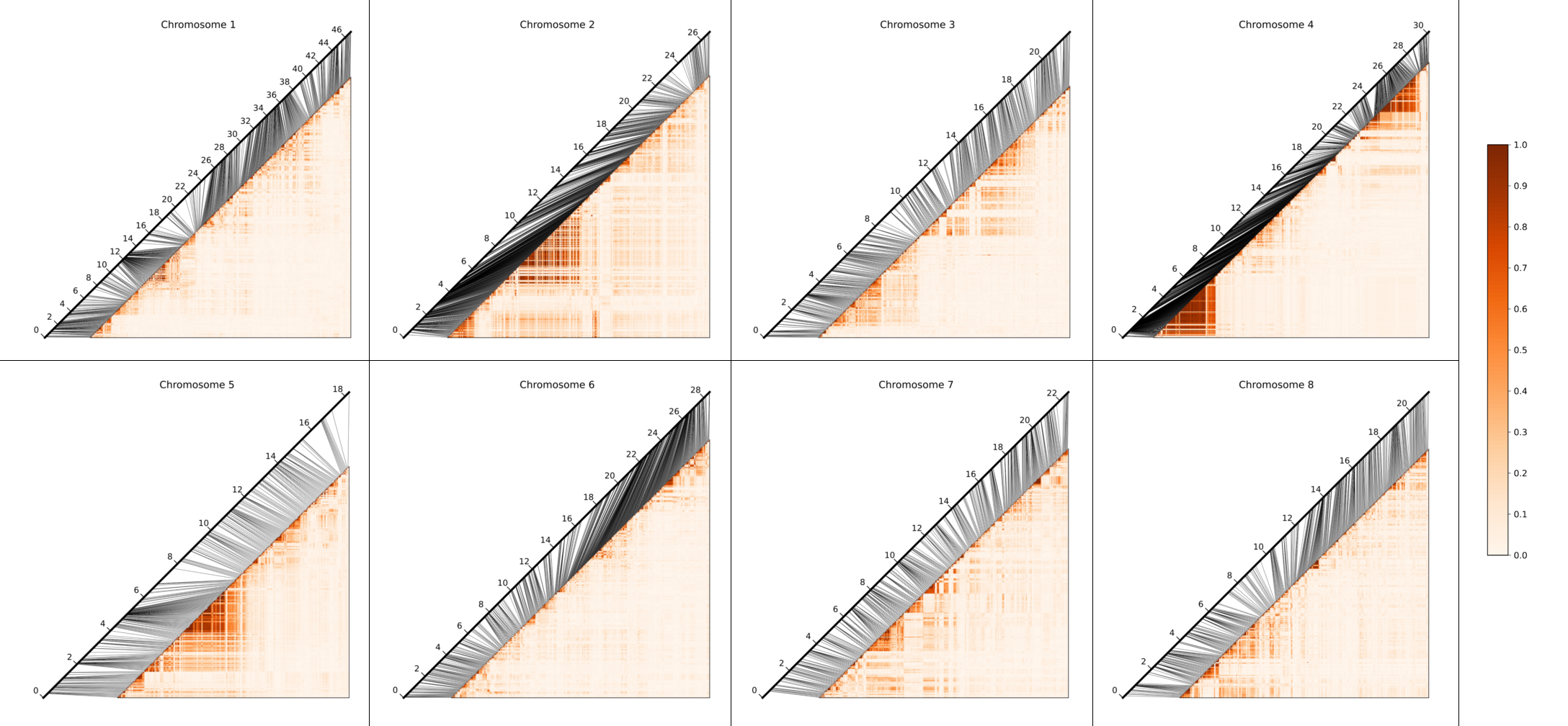

Supplement: S5 Fig — The diagonal black line indicates the physical position of the SNPs on the chromosome. (TIFF) [file pone.0136803.s005.tiff]

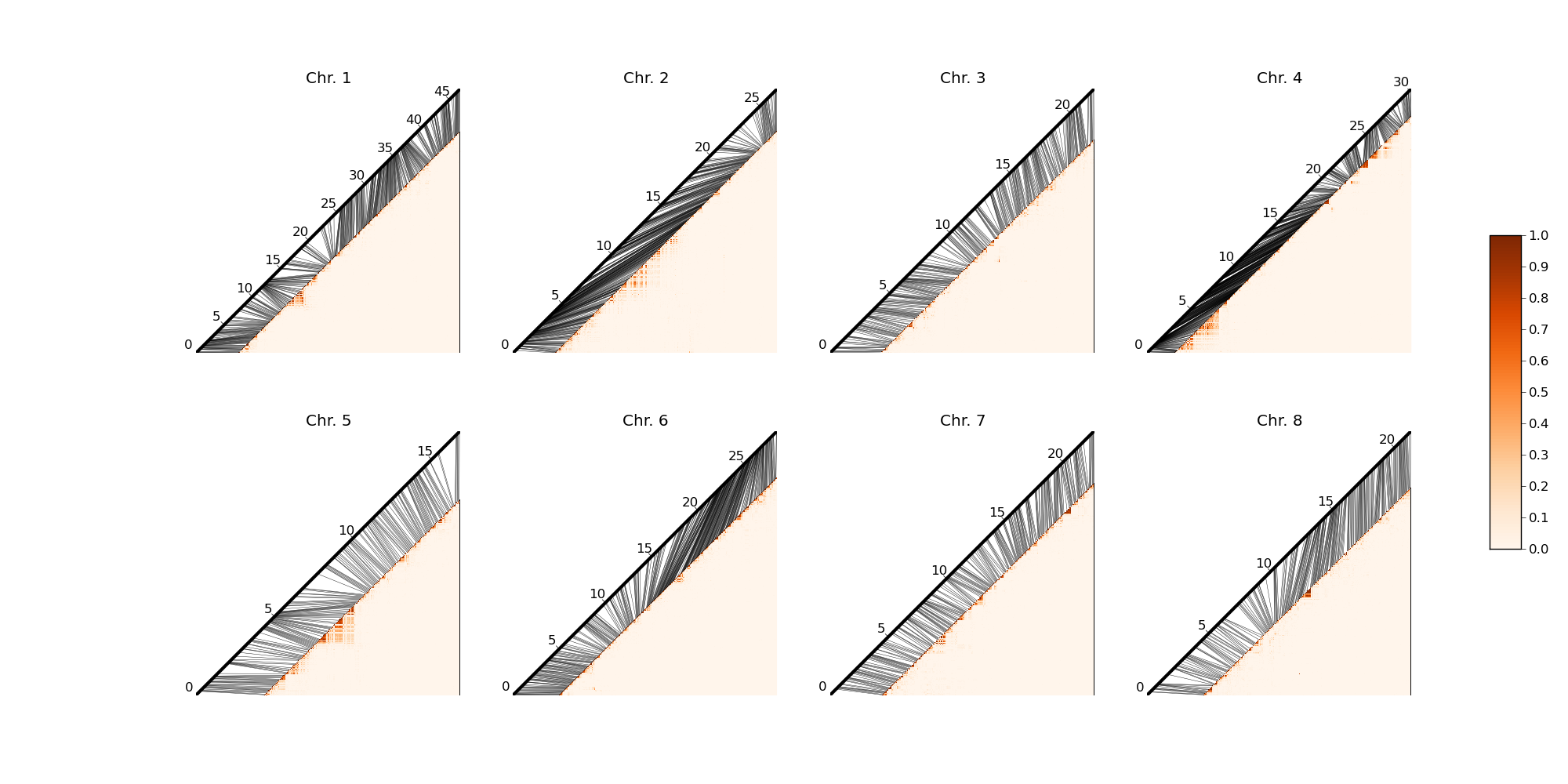

Supplement: S6 Fig — The diagonal black line indicates the physical position of the SNPs on the chromosome. (TIFF) [file pone.0136803.s006.tiff]
